# Organocatalytic Fluorogenic Synthesis of Chromenes

**DOI:** 10.1007/s10895-017-2049-7

**Published:** 2017-02-21

**Authors:** Mina Raeisolsadati Oskouei, Albert M. Brouwer

**Affiliations:** van ‘t Hoff Institute for Molecular Sciences, University of Amsterdam, PO Box 94157, 1090 GD Amsterdam, The Netherlands

**Keywords:** Chromene, Fluorescence spectroscopy, Organocatalysis, Fluorogenic reaction

## Abstract

Two fluorescent derivatives of 2-amino-3-carbonitrile-4H-chromene were synthesized by means of a fluorogenic Michael addition of dimedone to dicyano alkene labeled BODIPY derivatives. Different organocatalysts were used in different conditions to obtain compounds 3 and 4 in good yield (up to 65% and 85%) and moderate enantiomeric excess (51% and 41% ee, respectively). This work provides the first example of an enantioselective organocatalytic conversion combined with fluorogenesis.

**Electronic supplementary material:**

The online version of this article (doi:10.1007/s10895-017-2049-7) contains supplementary material, which is available to authorized users.

## Introduction

Chemical reactions that generate a bright fluorescent species are called “fluorogenic”. Such fluorescence turn-on is often used as a way to construct fluorescent markers for a particular substrate based on a reaction between the marker and a fluorophore precursor [1–6]. In our laboratory we have initiated a research program aimed at the study of organocatalytic reactions using fluorescence spectroscopy [7–9]. In this connection we explore fluorogenic reactions as a first step towards unravelling the details of the reaction mechanisms [10–12]. The present paper describes a novel fluorogenic organocatalytic reaction in which a fluorescent chromene derivative **3** is produced from the almost non-fluorescent precursor 1 [6, 13].

Chromene is a structural component in biologically active and natural compounds such as alkaloids, tocopherols, flavonoids, and anthocyanins [14–17]. Functionalized chromenes have attracted a lot of attention in the field of synthetic and medicinal chemistry [18–23]. Among the diverse chromene derivatives, 2-amino-4H-chromenes are reported as potential drugs in the treatment of human inflammatory TNFa-mediated diseases [24]. Cytotoxicity of 2-amino-3-carbonitrile-4H-chromene in human acute myeloid leukemia (AML) cell lines has been demonstrated. These compounds bind to the surface pocket of the cancer-implicated Bcl-2 protein and induce apoptosis or programmed cell death in follicular lymphoma B cells and leukemia HL-60 cells [25–27]. Luminescent labeling of cells is used for flow cytometry and microscopy [28–30]. The function of the cells can, however, be affected by the dye. Furthermore, some dyes cannot be used in combination with other dyes [31]. Having a broader spectrum of dyes provides more possibilities for researchers to overcome the limitations of the available ones. Especially, if the labeling agent is the drug itself, it will be possible to detect the components of the biological assemblies and imaging and flow cytometry at the same time. In this case, there is hope to find the mechanism of the interaction between the drug and the tumor cell to design more effective drugs. Thus, although it is not the primary aim of our study, the dye-labelled chromenes may find applications in biomedical research.

BODIPY (4,4-difluoro-4-bora-3a,4a-diaza-s-indacene) dyes are often the preferred choice for labelling applications. They are relatively nonpolar and the chromophore is electrically neutral. These properties tend to minimize dye-induced perturbation of the functionality of the labelled species [32–35].

**Scheme 1:**
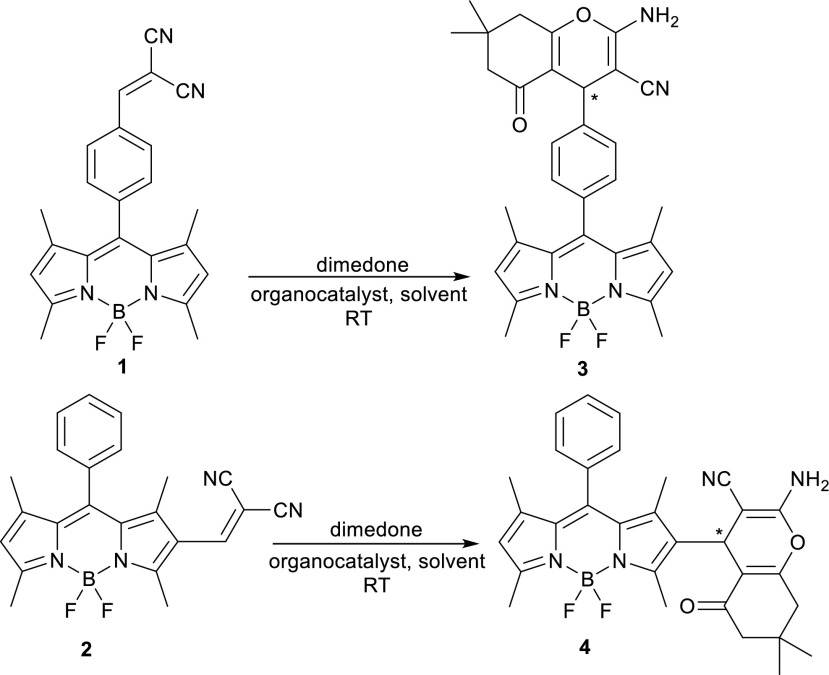
Synthesis of 2-amino-3-carbonitrile-4H-chromenes **3** and **4**

In the present work we couple two dicyano alkene derivatives of BODIPY (compounds **1** and **2**) [6, 13, 36–38], with dimedone to produce the corresponding fluorescent chromenes (Scheme 1). Compounds **1** and **2** have been used as a fluorescent turn-on and turn-off probes, respecetively, for the detection of cyanide in solution [37, 38]. To the best of our knowledge there is no report about the application of compounds **1** and **2** in organocatalytic synthetic reactions. In this article we present the main results of the first usage of these compound in organocatalytic Michael addition reactions, which are followed up in situ by a ring closure leading to chromenes.

In compounds **1** and **2** the BODIPY skeleton is responsible for the fluorescence. In compound **1** the fluorescence is strongly quenched by a photo induced electron transfer mechanism [37]. The BODIPY part of the molecule acts as an electron donor [39, 40], the dicyanoalkene as the electron acceptor. In this compound the two units are not effectively conjugated because the 8-phenyl substituent is almost orthogonal to the BODIPY [40]. In compound **2**, on the other hand, the fluorescence is not quenched. In this case, the dicyanoalkene group is directly conjugated with the BODIPY unit, and the excited state has mostly a delocalized π-π* character [36].

The Michael addition to the double bond of the dicyano alkene in **1** turns on the fluorescence because this effectively removes the electron acceptor unit. This phenomenon allows us to use fluorescence spectroscopy to follow the addition of the nucleophile, deprotonated dimedone in this case, to form compound **3** after a ring closure step.

**Scheme 2:**
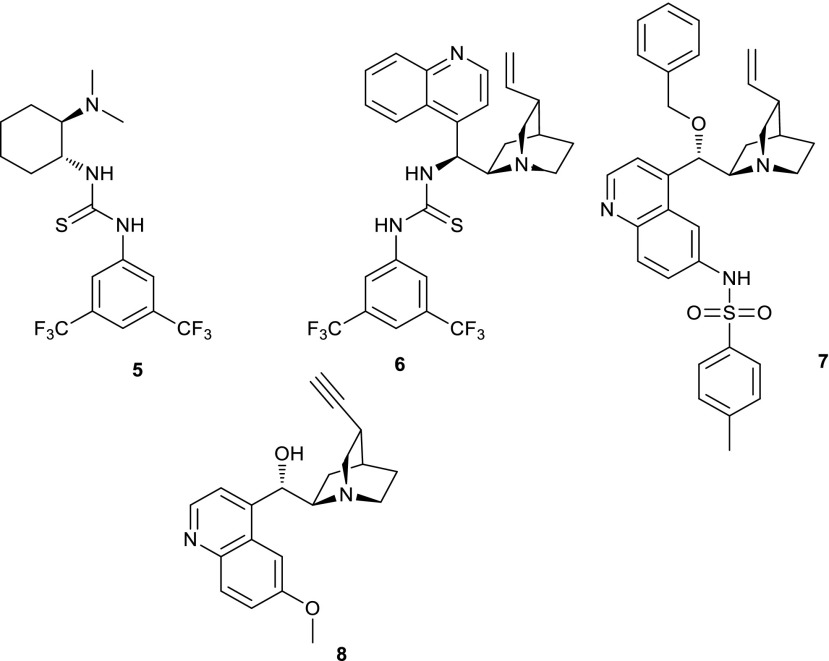
Organocatalysts used

The ability of hydrogen bond forming catalysts to speed up and control the enantioselectivity of the Michael addition reactions has been amply demonstrated. Among the many available catalysts, we selected catalysts **5–8** which have been reported to promote Michael additions in high yield and enantioselectivity (Scheme 2) [41, 42].

In these catalysts, the amine group provides the required basicity to produce the nucleophilic dimedone anion and the hydrogen bond donating groups can activate the Michael acceptor by hydrogen bonding to the cyano groups.

## Results and Discussion

The reaction between dicyanoalkene-BODIPY **1** and dimedone was performed using different catalysts in dichloromethane (DCM) at room temperature (Table 1).

**Table 1:** Reaction between dimedone and compound **1** in the presence of different catalysts in DCM at room temperature

Catalyst (10 mol%)	**5**	**6**	**7**	**8**
ee (%)	44	42	20	10
Yield (%)	80	85	73	70

The enantioselectivity in the presence of catalyst 6 is similar to that obtained with catalyst **5** and is low in the presence of catalysts **7** and **8**. The result of the reactions between dicyanoalkene-BODIPY **1** and dimedone in the presence of catalyst **5** showed catalysis of the reaction in both polar and non-polar solvents at room temperature (Table 2). Reaction in DCM and toluene at room temperature provided the product with 42–44% enantiomeric excess (ee). The reaction in tetrahydrofuran (THF) was not enantioselective. The progress of the reaction was followed in DCM at different temperatures (Table 2). Decreasing the temperature slows down the reaction, but the enantiomeric excess is higher.

**Table 2:** Reaction between dimedone and compound **1** in the presence of catalyst **5** (10 mol%**)**

Solvent	Toluene	DCM	DCM	DCM	THF
Temperature (°C)	25	25	0	-20	25
ee (%)	42	44	44	51	0

We applied similar conditions for the reactions between compound **2** and dimedone in the presence of the different catalysts (Table 3).

**Table 3:** Reaction between dimedone and compound **2** in the presence of different catalysts at room temperature

Catalyst (10 mol%)	**5**	**5**	**6**	**7**	**8**
Solvent	Toluene	DCM	DCM	DCM	DCM
ee (%)	27	34	41	12	34
Yield (%)	65	68	72	68	65

In compound **2** conjugation of the double bond of the dicyano alkene group with the pyrrole moiety of the BODIPY decreases the nucleophilicity of this group. As a result, the reaction with dimedone is slower for compound **2** than for compound **1**. The structural assignments are provided in the Supporting Information.

We determined photophysical properties of the pure reactants and products by means of absorption and fluorescence spectroscopy, and time-resolved fluorescence [43–45]. The results are summarized in Table 4. The absorption spectra are all similar, as expected, with small red shifts for **2** and **4**, in which the BODIPY core is substituted. The absorption coefficients and radiative rate constants are similar, and characteristic for the BODIPY chromophore.

**Table 4:** Photophysical parameters of compounds **1**, **2**, **3** and **4** in DCM

Compound	*λ*_*max, abs*_^a^ (nm)	*ε* ^b^ (10^3^ L mol^−1^ cm^−1^)	*λ*_*em*_^c^ (nm)	*ϕ*_*f*_^d^	*τ* ^e^ (ns)	*k*_*f*_^f^ (s^−1^)	*k*_*nr*_^g^ (s^−1^)
**1**	505	75	517	0.025	0.01(0.57); 1.4 (0.30); 3.1 (0.13)		
**2** ^h^	516 (514)	77 (55)	532 (543)	0.54 (0.45)	3.08	1.7 × 10^8^	1.5 × 10^8^
**3**	500	74	511	0.45	3.50	1.3 × 10^8^	1.6 × 10^8^
**4**	512	78	523	0.61	3.94	1.6 × 10^8^	1.0 × 10^8^

^a^Absorbance maximum, ^b^Molar absorption coefficient, ^c^Emission maximum, ^d^Quantum yield, ^e^Decay time; for **1** the three time constants are given with amplitudes in parentheses, ^f^Fluorescence rate constant *k*_*f*_ *= ϕ*_*f*_ */τ*, ^g^Non-radiative rate constant *k*_*nr*_ *= τ* ^*−1*^ *- k*_*f*_, ^h^ Literature values from ref. 11c are given in parentheses

The fluorescence decays of compounds **2**, **3**, and **4** are described very well by a mono-exponential model. In the case of compound **1**, however, we observed a tri-exponentially decaying intensity with a time constant of ~10 ps for the major fraction, corresponding to the strongly quenched fluorescence. The time resolution of our set-up is insufficient to resolve this properly, so the real time constant may be smaller than 10 ps. A slow decay component is present with a time constant similar to that of the other BODIPY derivatives and may be due to a minor impurity in the sample. A third component with an intermediate decay time is clearly present, however. Further research will be needed to ascertain its origin.

We applied fluorescence spectroscopy to follow the progress of the Michael reaction. In order to be able to measure the fluorescence of the reaction mixture directly, an HPLC pump was used to circulate the solution through a microcuvette in the sample compartment of the fluorescence spectrometer. Because the optical path length is short, internal filter effects are less important. By using the circulation pump we can work with practically manageable quantities of material and a reaction volume of 4 mL, and provide for continuous mixing of the reagents.

**Fig. 1:**
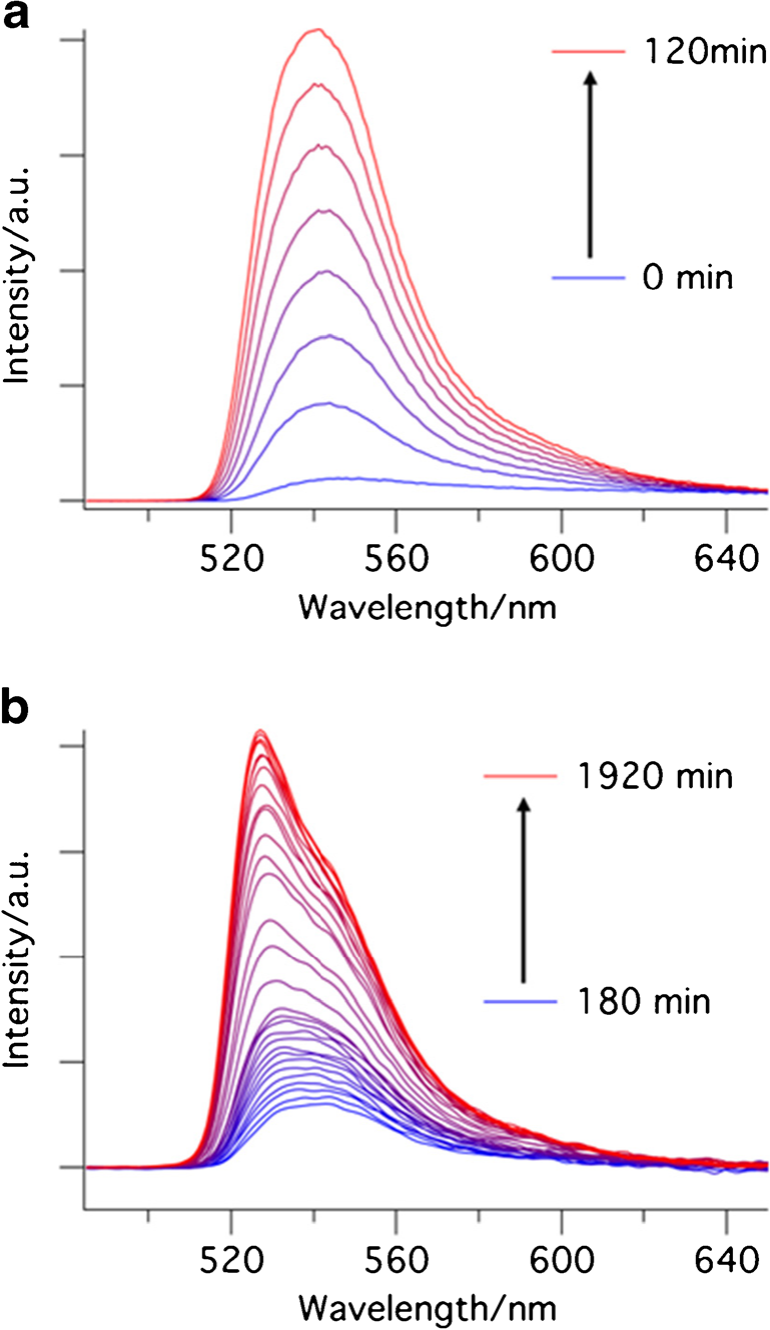
Emission spectra (λ_ex_ = 478 nm) of the mixture of the reaction between dimedone and compound 1 in the presence of catalyst **5** in DCE at room temperature. **a** during the first two hours, **b** during the later stages of the reaction

The emission spectrum of the mixture was measured every 30 min for 32 h. In order to decrease the error due to evaporation of the solvent and changing the concentration of the mixture, we used the less volatile dichloroethane (DCE) as the solvent instead of DCM. The emission of the solution of compound **1** in DCE was measured. Then, catalyst **5** and dimedone were added to the solution. The increase of the intensity of fluorescence, already clearly visible after 5 min, shows formation of the product. This increase slows down after 25 h (Fig. 1).

**Scheme 3:**
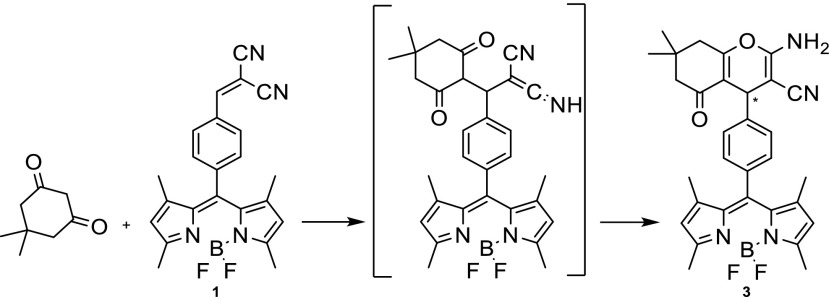
Mechanism of formation of compound **3**

It is evident that the shape of the spectrum changes during the course of the reaction. Initially, the product spectrum is broad and peaks at ~540 nm (Fig. 1(a)), later it shows a pronounced peak at 532 nm (Fig. 1(b)). We tentatively attribute this change to the presence of a distinct intermediate, which initially builds up, and then decays as the final product is formed in a cyclization reaction (See Scheme 3).

**Fig. 2:**
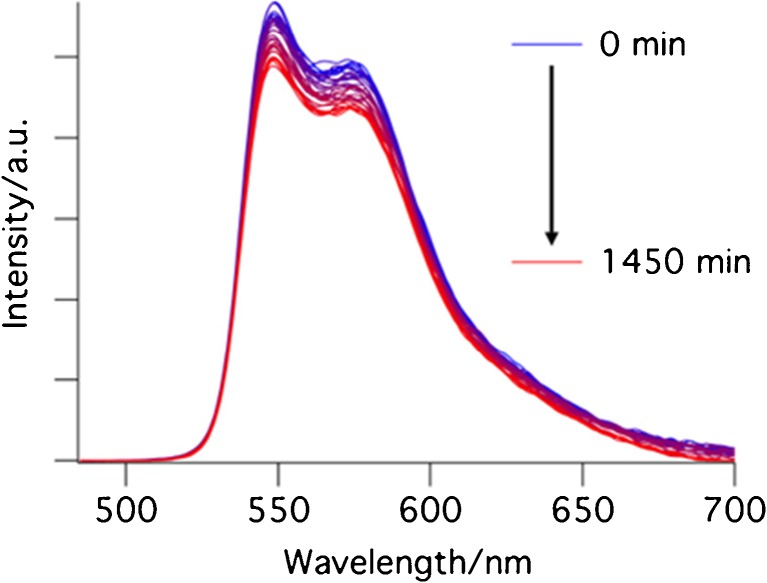
Emission spectra of the mixture of the reaction between dimedone and compound **2** in the presence of catalyst **5** in DCE at room temperature (λ_ex_ = 478 nm)

In contrast to compound **1** [6, 13, 37], its isomer **2** is strongly fluorescent [36, 38]. The direct interaction of the dicyano alkene group with the pyrrole moiety increases the length of the conjugated system, which leads to red shifted absorption and emission spectra, but also to lower reactivity because the electron rich BODIPY donates some electron density to the Michael acceptor group. As a result, the reaction of compound **2** with dimedone is clearly slower (Fig. 2) than that of **1**.

**Fig. 3:**
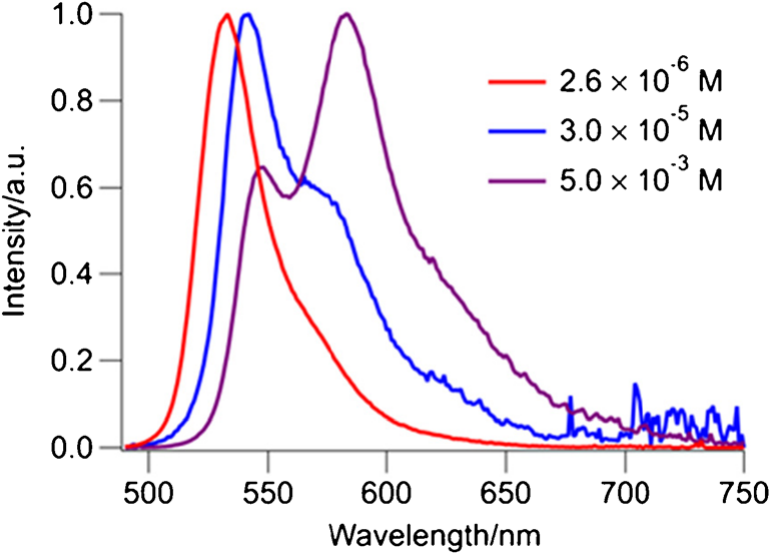
Emission spectra of compound **2** in dichloromethane at different concentrations (λ_ex_ = 478 nm)

We note that the shape and the position of the emission spectrum of compound **2** and its reaction product in this experiment are notably different from the spectra at low concentrations that were used to determine the photophysical properties. The red-shifted and broadened spectra are due to the higher concentrations used in the reaction mixture. At higher concentrations the second shoulder appears at longer wavelengths. The intensity of this new shoulder increases by increasing the concentration. This change can arise from aggregation of the chromphores (Fig. 3). During the reaction leading to product 4, we observe only a small change in the intensity of the emitted light and no change of the spectral shape.

## Conclusion

This work provides a simple method to synthesize labeled chromenes with good yields and enantiomeric excess, and introduces fluorescence spectroscopy as a powerful tool to follow the reaction of the fluorogenic substrate **1**. An intermediate of the two-step reaction could be detected by its fluorescence spectrum that is different from that of the product. The fluorescence of products **3** and **4** allows these compounds to be screened using imaging methods and opens a new avenue for the study of the efficiency of these compounds in the treatment of diseases.

### Experimental

#### Materials and Methods

All commercially available reagents and solvents were used as received. Catalyst **5** was obtained from Sigma Aldrich. Catalysts **6** and **8** were synthesized following the literatures procedures [46, 47]. Catalyst **7** was prepared in the organic synthesis group of the University of Amsterdam [48]. Flash column chromatography was carried out using silica gel 60A, 0.040–0.063 mm. Commercially available pre-coated TLC plastic sheets (Silica gel 60 F254) were used for thin layer chromatography (TLC). Preparative TLC was carried on commercially available pre-coated TLC glass plates (PLC Silica gel 60 F254, 1 mm). A UV lamp (254 or 366 nm) was used for visualization. Chiral HPLC was performed using a Shimadzu LC-20 AD liquid chromatograph equipped with SPD-M20A diode array detector and chiral OD-H column. 20% Isopropanol in heptane was used as the eluent. 1H and 13C NMR spectra were recorded on a Bruker Avance 400 spectrometer and analyzed using the MestReNova v 7.1.2 (Mestrelab Research S.L.) software. Signal positions were recorded in δ ppm with the abbreviations s, d, dd and m denoting singlet, doublet, doublet of doublets and multiplet respectively. All 1H NMR chemical shifts are referenced to SiMe_4_ as an external standard (0.00 ppm). All 13C NMR chemical shifts in CDCl_3_ were referenced to the residual solvent peak at 77.00 ppm but are reported vs. tetramethylsilane. All coupling constants, J, are quoted in Hz. Infra-red spectra were recorded on a Bruker IR spectrometer model α-Platinum ATR using neat solid samples. Mass spectra were collected on an AccuTOF LC, JMS-T100LP Mass spectrometer (JEOL, Japan). The measurement conditions were as follows: Positive-ion mode; Needle voltage 2000 V, Orifice 1 voltage 90 V, Orifice 2 voltage 9 V, Ring Lens voltage 22 V. Ion source temperature 30 °C, spray temperature − 20 °C. Flow injection with a flow rate of 0.01 ml/min. The UV-Vis absorption spectra were recorded on a double beam Shimadzu UV-2700 spectrophotometer. Fluorescence excitation and emission spectra of the compounds were recorded using a SPEX Fluorolog 3–22 fluorimeter. The concentrations were chosen to have A = 0.1 in a 1 cm cell at the excitation wavelength (c ≈ 10^−6^ M). A Gilden Photonics FluoroSense-M series spectrometer equipped with two double monochromators was used to follow the reactions. A Bischoff HPLC pump was used to circulate the solution. DCM dye (4-(dicyanomethylene)-2-methyl-6-(4-dimethylaminostyryl)-4H–pyran) was used as the reference to determine the fluorescence quantum yields (ɸ_f_ = 0.43) [49]. The measurement of fluorescence decay times was performed as described in reference [9]. The excitation wavelength was λ_ex_ = 478 nm. Decay curves were fitted to a sum of exponential decays using a non-linear least-squares routine implemented in Igor Pro 6.3 (Wavemetrics, Inc.). In all cases the χ^2^ value was <1.1, indicating excellent fits.

#### Synthesis of **1**

Compound **1** (8-(4-(2,2-dicyanovinyl)phenyl)-4,4-difluoro-1,3,5,7-tetramethyl-4-bora-3a,4a–diaza-s-indacene) was prepared according to references [6, 13]. Analytical data are in agreement with the literature.

#### Synthesis of **2**

Compound **2** (6-(2,2-dicyanovinyl)-8-phenyl-4,4-difluoro-1,3,5,7-tetramethyl-4-bora-3a,4a-diaza-s-indacene) was prepared according to reference [37]. Analytical data are in agreement with the literature.

#### Synthesis of **3**

Compound **1** (40 mg, 0.1 mmol) was dissolved in 4 ml solvent (see Table 2), dimedone (15.4 mg, 0.11 mmol) and organocatalysts (0.01 mmol) were added. The mixture was stirred at room temperature for 24 h. The product was purified using flash column chromatography (25% EtOAc/petroleum ether). 1H NMR (400 MHz, CDCl_3_): δ (ppm) = 7.43 (d, 2H, J = 8 Hz, ArH), 7.23 (d, 2H, J = 8 Hz, ArH), 5.98 (s, 2H, CH-pyrrole), 4.61 (s, 2H, NH_2_), 4.52 (s, 1H, CH), 2.56 (s, 6H, CH_3_), 2.48 (AB pattern, 2H, ΔδAB = 0.09 ppm, J = 20 Hz, CH_2_), 2.23 (AB pattern, 2H, ΔδAB =0.16 ppm, J = 16 Hz, CH_2_), 1.35 (s, 6H, CH_3_), 1.14 (s, 3H, CH_3_), 0.98 (s, 3H, CH_3_). 13C NMR (100 MHz, CDCl_3_): 195.24, 161.11, 157.46, 154.18, 144.04, 143.09, 133.59, 128.52, 127.87, 121.02, 117.99, 113.85, 99.81, 62.89, 50.45, 40.48, 35.35, 31.92, 28.97, 26.72, 14.39, 14.09. IR: ν (cm^−^1): 3450, 3338, 3220, 2954, 2192, 1676, 1597, 1541, 1507, 1467, 1360, 1305, 1213, 1190, 1038, 971. High resolution mass calculated for (C_31_H_31_BF_2_N_4_O_2_): 540.25081, Found: 540.24910.

#### Synthesis of **4**

Compound **2** (0.1 mmol, 40 mg) was dissolved in 4 ml solvent (see Table 3), dimedone (0.11 mmol, 15.4 mg) and organocatalyst (0.01 mmol) were added. The mixture was stirred at room temperature for 48 h. The product was purified using flash column chromatography (25% EtOAc/ petroleum ether). 1H NMR (400 MHz, CDCl_3_): δ (ppm) = 7.49 (m, 3H, ArH), 7.33 (m, 2H, ArH), 5.98 (s, 1H, CH-pyrrole), 4.51 (s, 2H, NH_2_), 4.43 (s, 1H, CH), 2.56 (s, 6H, CH_3_), 2.40 (AB pattern, 2H, ΔδAB =0.06 ppm, J = 16 Hz, CH_2_), 2.23 (AB pattern, 2H, ΔδAB =0.03 ppm, J = 16 Hz, CH_2_), 1.58 (s, 6H, CH_3_), 1.11 (s, 3H, CH_3_), 1.08 (s, 3H, CH_3_). 13C NMR (100 MHz, CDCl_3_): 195.75, 161.08, 157.14, 155.14, 143.04, 135.07, 131.48, 129.89, 128.94, 128.08, 121.09, 118.23, 112.19, 61.20, 50.54, 47.04, 40.41, 31.99, 28.40, 28.00, 25.61, 14.47, 12.48, 11.39, 8.62. IR: ν (cm^−^1): 3338, 3175, 2957, 2925, 2191, 1680, 166s4, 1598, 1537, 1512, 1465, 1358, 1309, 1191, 1158, 976. Mass calculated for (C_31_H_31_BF_2_N_4_O_2_) + CH_3_CN + Na: 604.26713, Found: 604.26829.

#### Monitoring the Michael Reaction between BODIPY-Dicyanoalkene (Compound ***1*** or Compound ***2***) and Dimedone in the Presence of the Catalysts

In these reactions, (0.02 mmol) BODIPY-Dicyanoalkene was dissolved in 4 ml 1,2-dichloroethane. The solution was circulated through a 3 mm path length quartz flow cuvette. The emission spectrum of the solution was measured. Then, dimedone (0.03 mmol) and catalyst (0.002 mmol) were added. The emission spectra of the solution were automatically measured every 30 min. The excitation wavelength was λ_ex_ = 478 nm.

## Electronic supplementary material

ESM 1 (PDF 1474 kb)
