# Supplementary material for: Organocatalytic Fluorogenic Synthesis of Chromenes
Source: J Fluoresc. 2017 Feb 21;27(3):1141–7. doi: 10.1007/s10895-017-2049-7 (PMC5393152; doi:10.1007/s10895-017-2049-7)
Supplement: Supplementary file 1 — (PDF 1474 kb) [file 10895_2017_2049_MOESM1_ESM.pdf]

Organocatalytic fluorogenic synthesis of chromenes

Mina Raeisolsadati Oskouei, Albert M. Brouwer
University of Amsterdam, van ‘t Hoff Institute for Molecular Sciences, PO Box 94157, 1090 GD, Amsterdam, The Netherlands

Contents of Supporting Information

1. IR spectra 2
2. NMR spectra 3
3. Mass spectra 5
4. Absorption, fluorescence excitation, and emission data 6

1. IR spectra

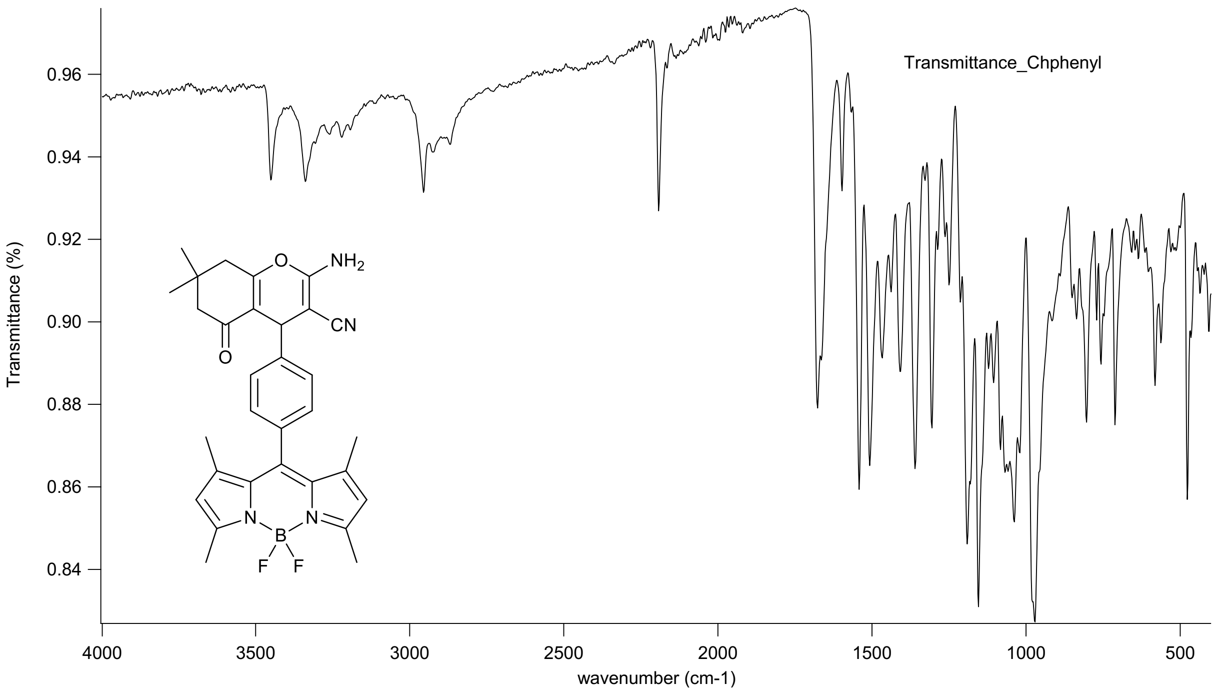

Figure S1. IR spectrum of compound **3**.

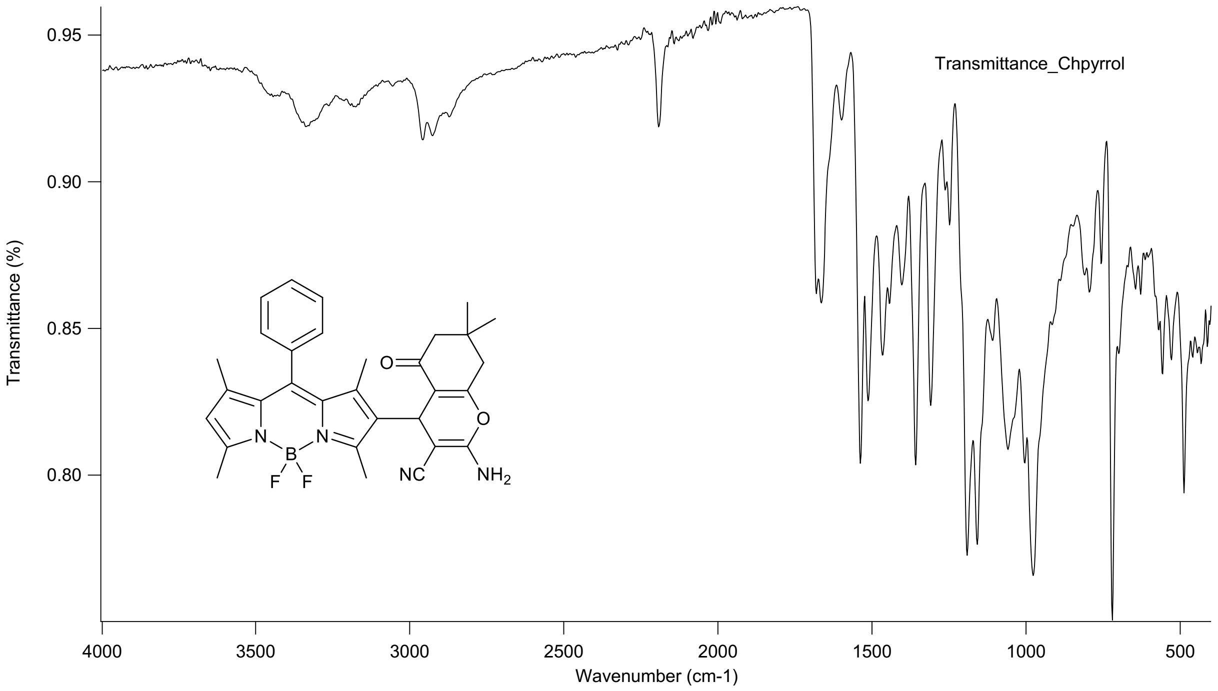

Figure S2. IR spectrum of compound **4**.

2. NMR spectra

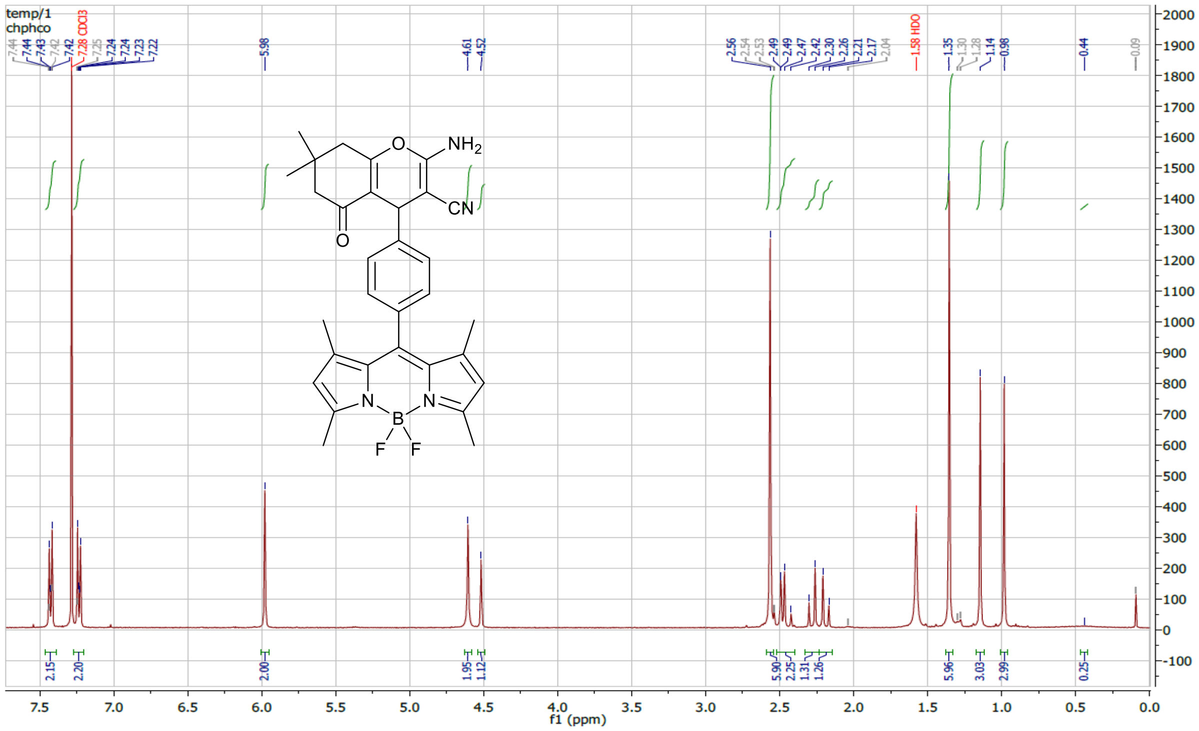

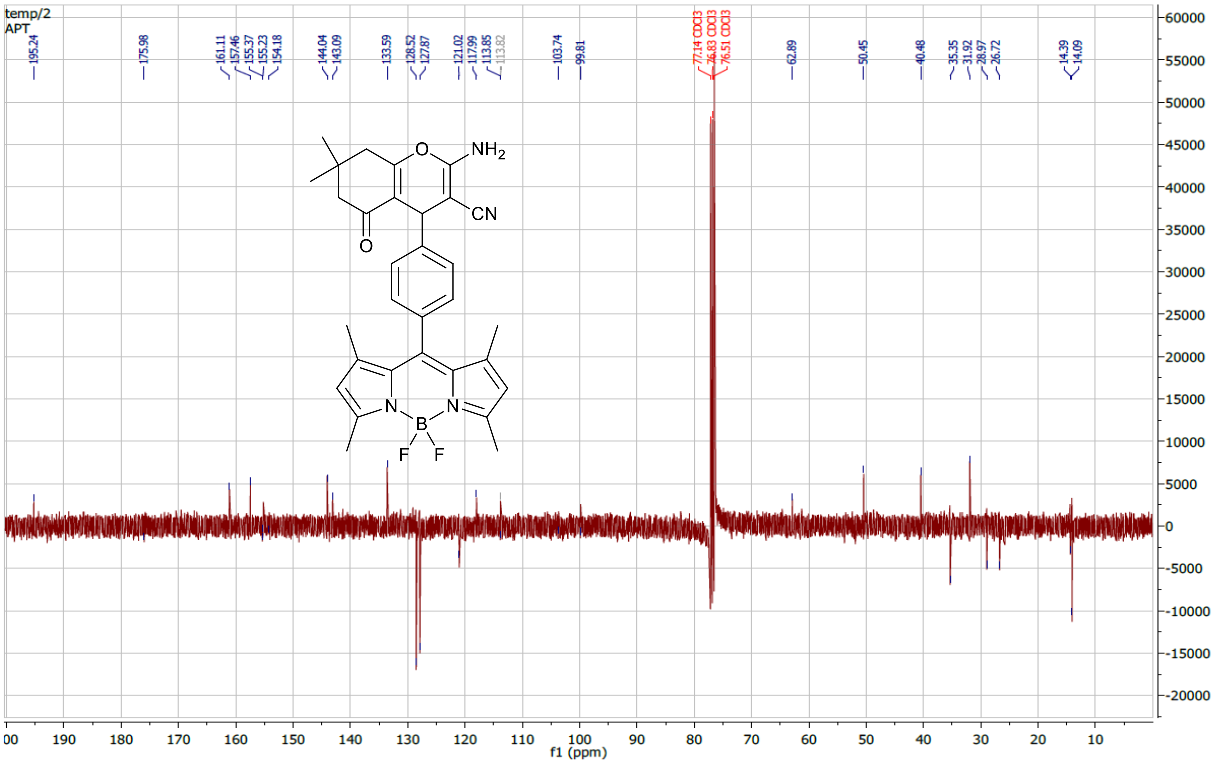

Figure S3. ^{1}H and ^{13}C NMR spectra of compound **3**.

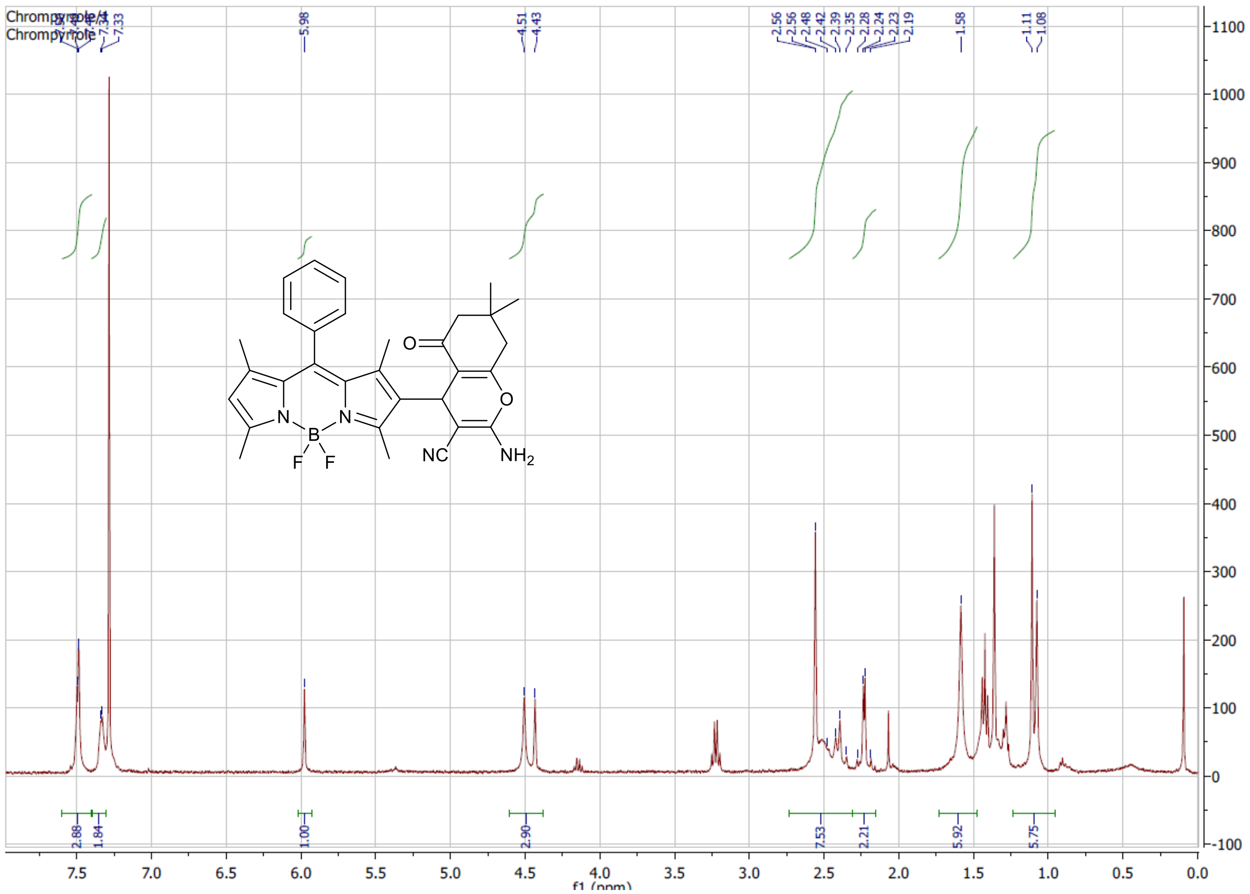

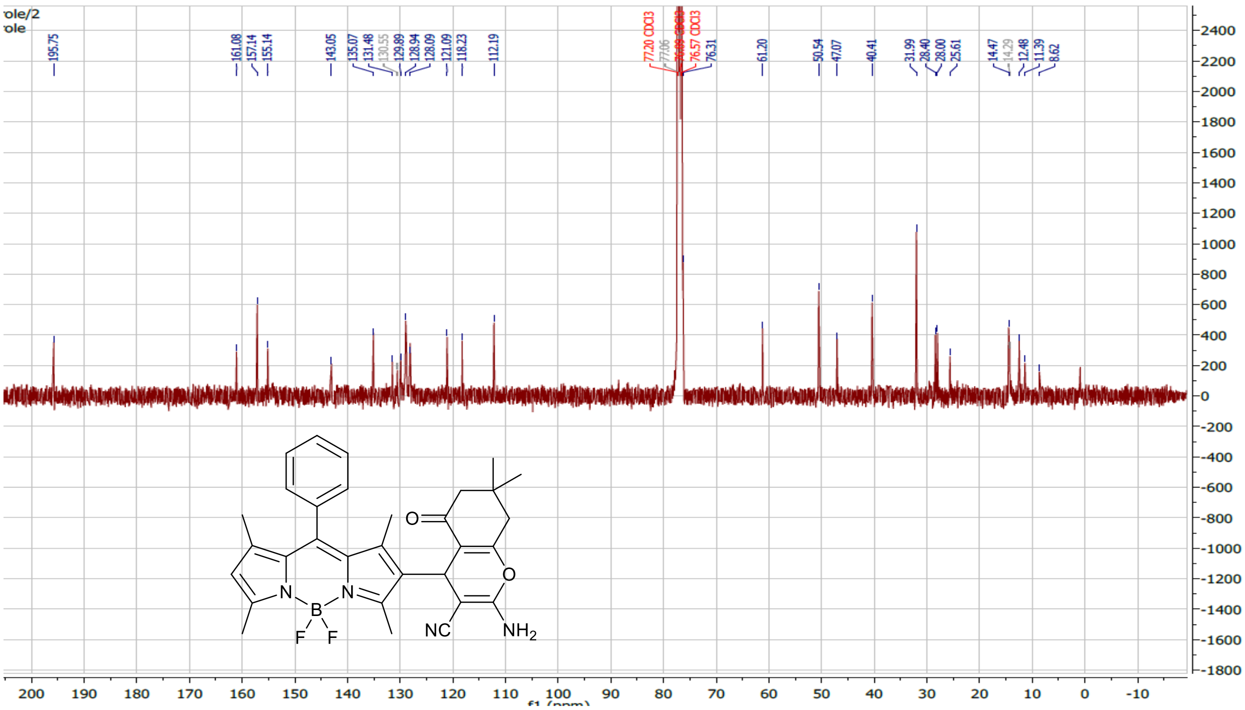

Figure S4. ^{1}H and ^{13}C NMR spectra of compound **4**.

3. Mass spectra

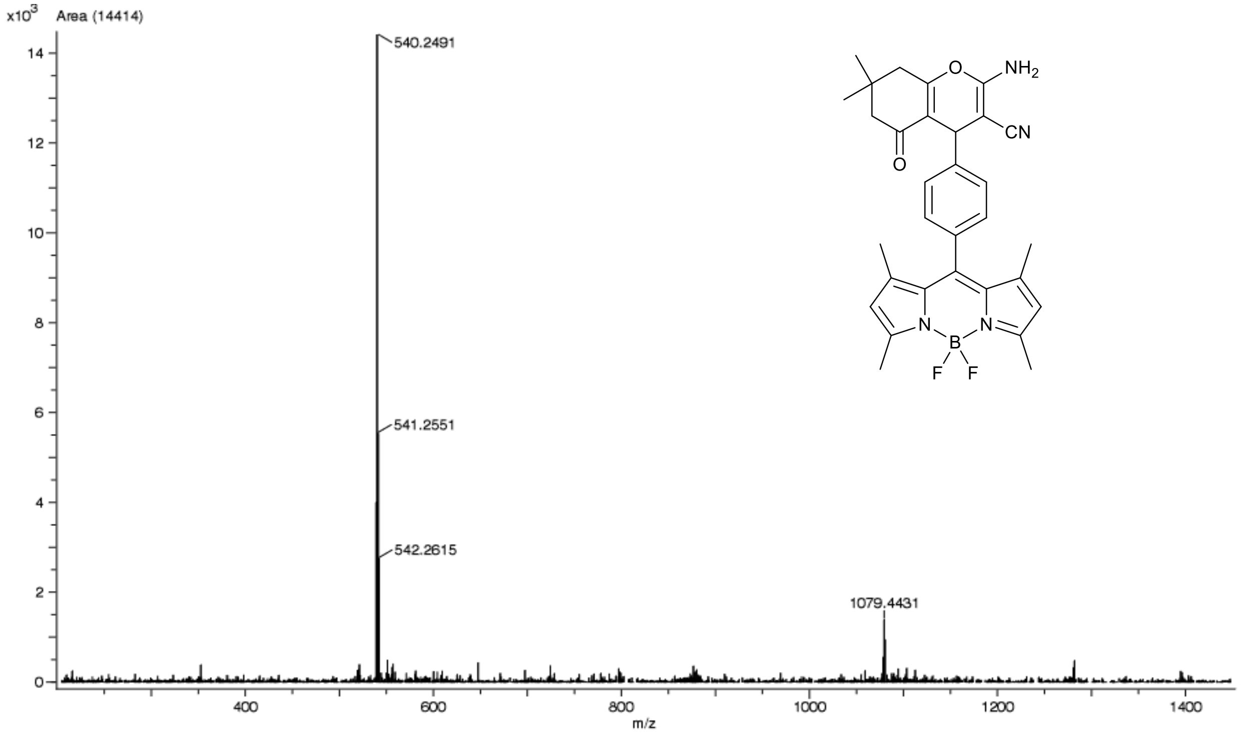

Figure S5. Mass spectrum of compound **3**.

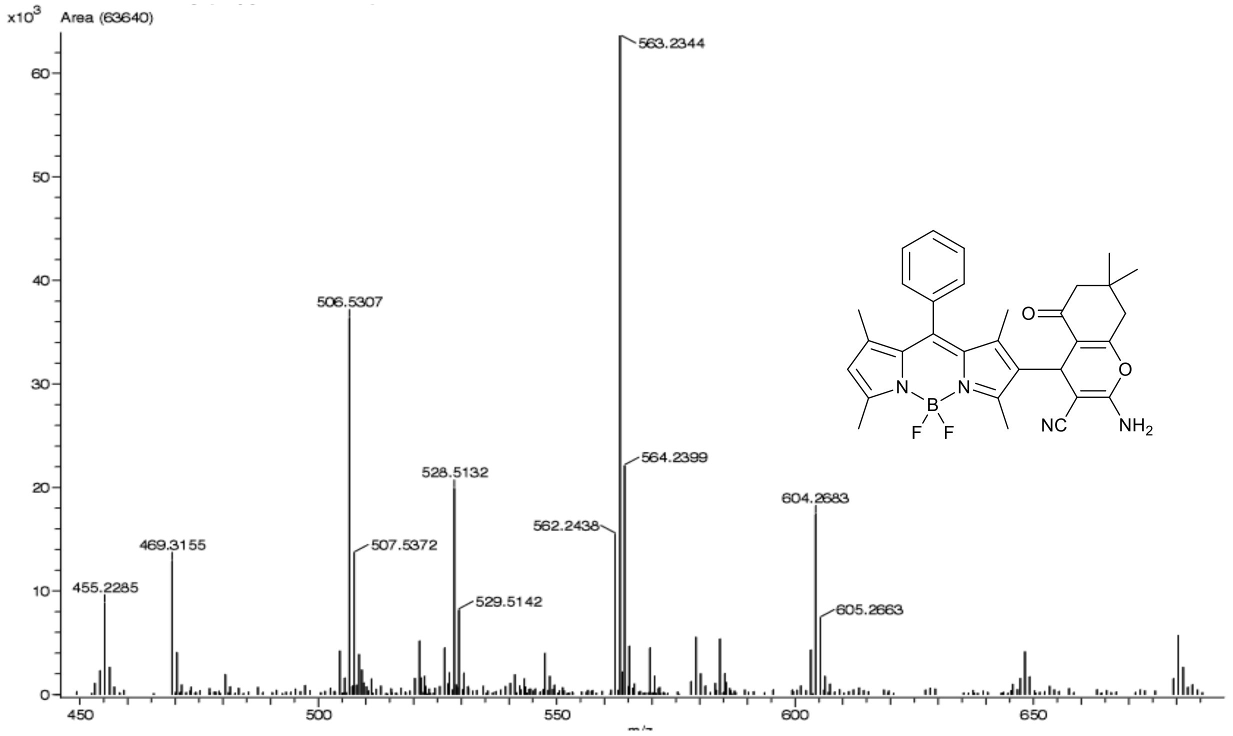

Figure S6. Mass spectrum of compound **4**.

4. Absorption, fluorescence excitation, and emission data

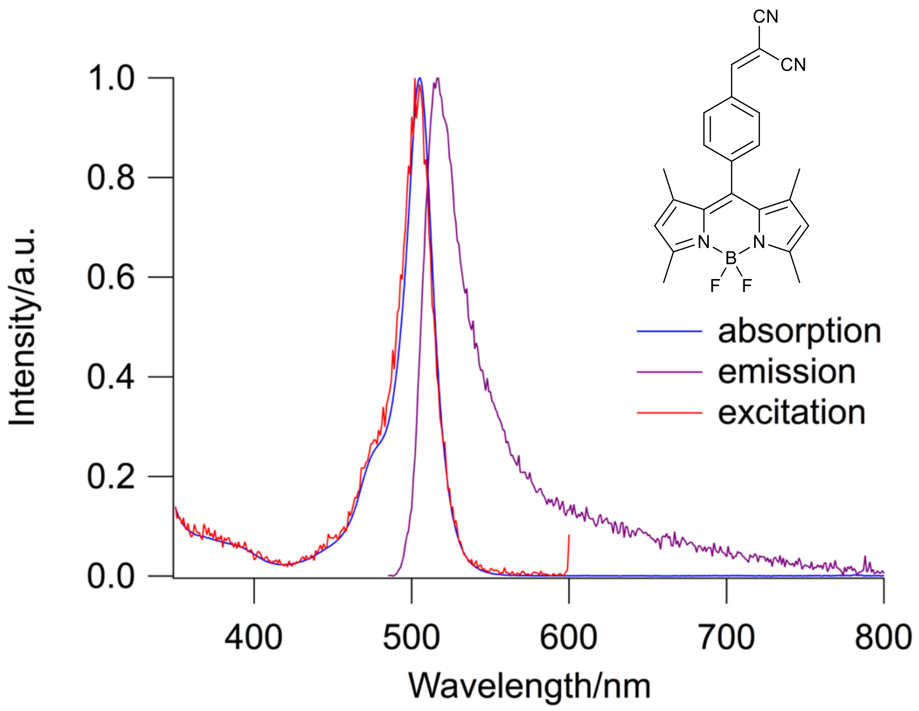

Figure S7. Absorption, fluorescence (λ_{ex} = 478 nm) and fluorescence excitation spectra (λ_{em} = 605 nm) of compound **1** in DCM**.**

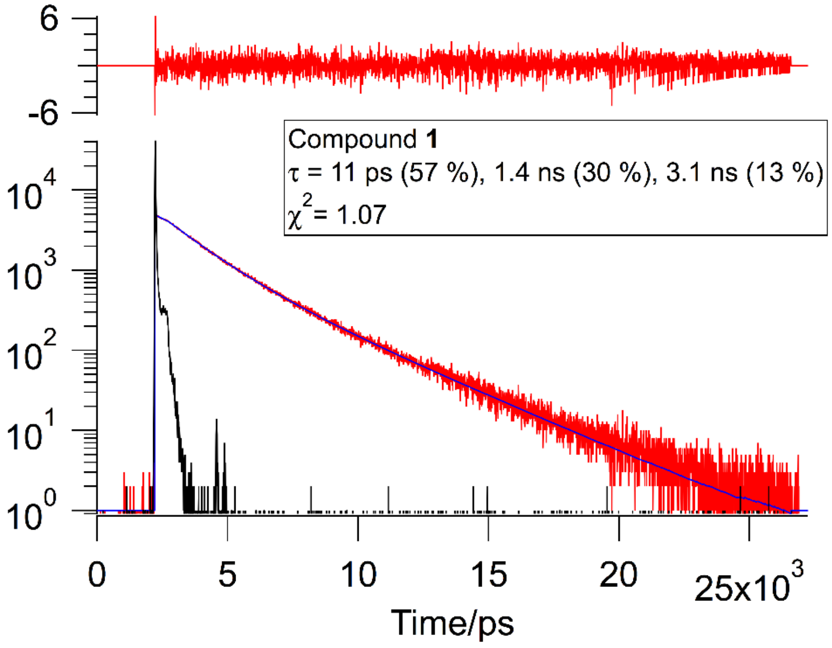

Figure S8. Time resolved fluorescence of compound **1** in DCM. The blue line is the fitted curve. The instrument response function is shown in black.

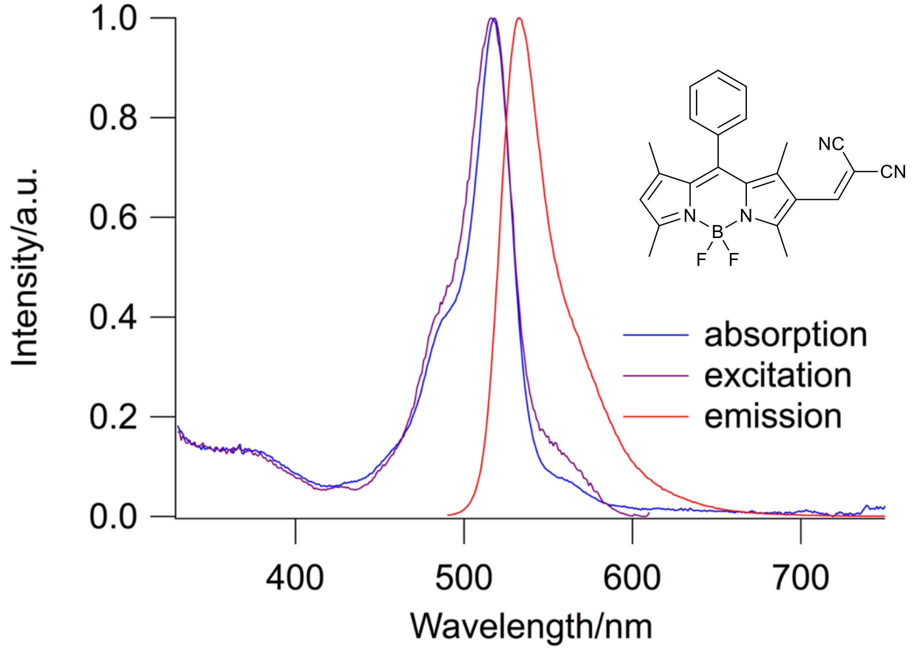

Figure S9. Absorption, fluorescence (λ_{ex}= 485 nm) and fluorescence excitation spectra (λ_{em} = 625 nm) of compound **2** in DCM.

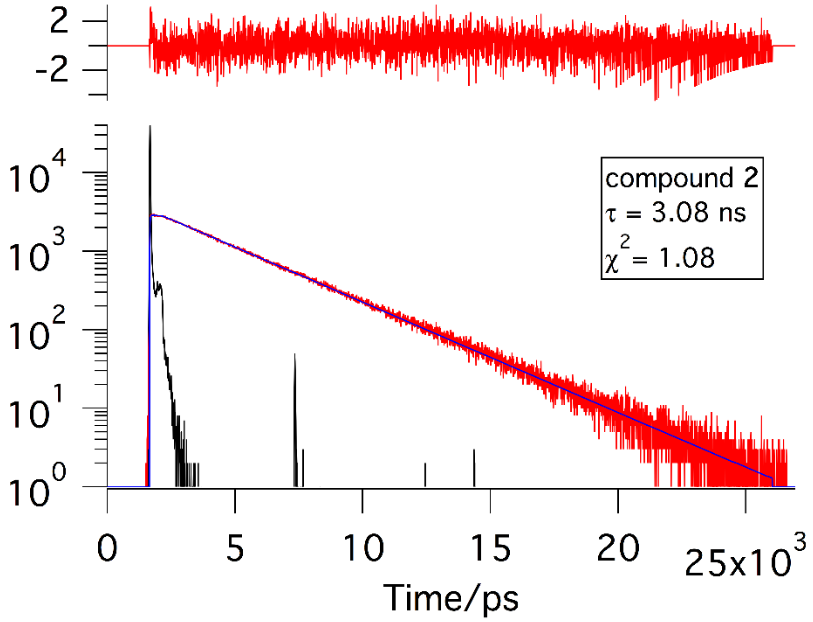

Figure S10. Time resolved fluorescence of compound **2** in DCM. The smooth blue line is the fitted curve. The instrument response function is shown in black.

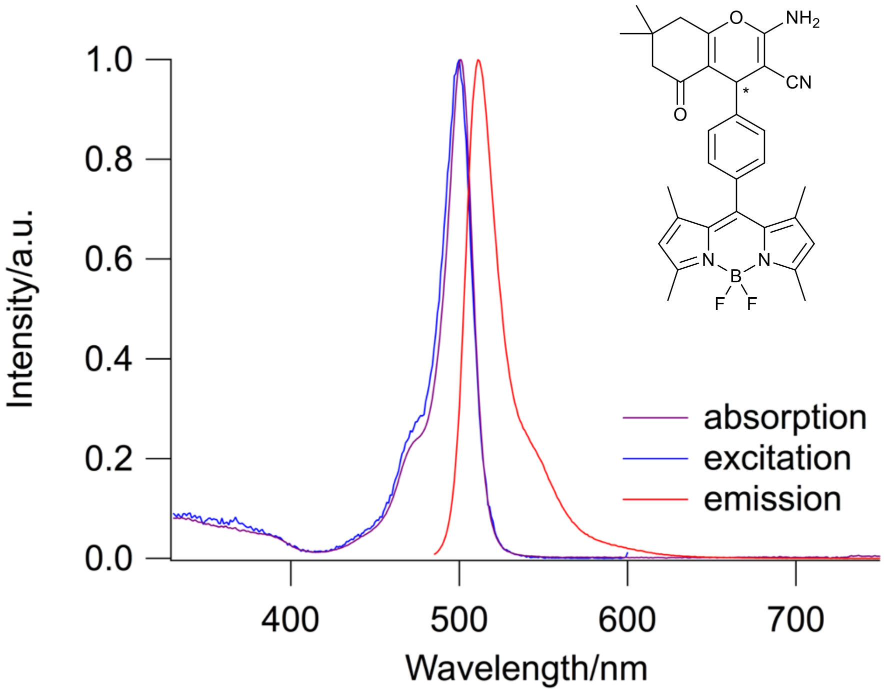

Figure S11. Absorption, fluorescence (λ_{ex} = 478 nm) and fluorescence excitation spectra (λ_{em} = 605 nm) of compound **3** in DCM.

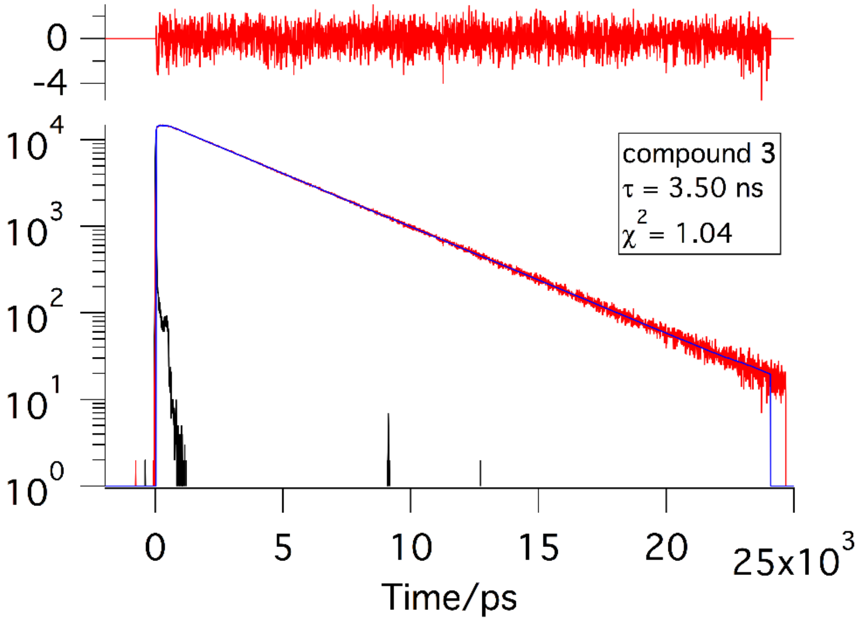

Figure S12. Time resolved fluorescence of compound **3** in DCM. The blue line is the fitted curve. The IRF is shown in black.

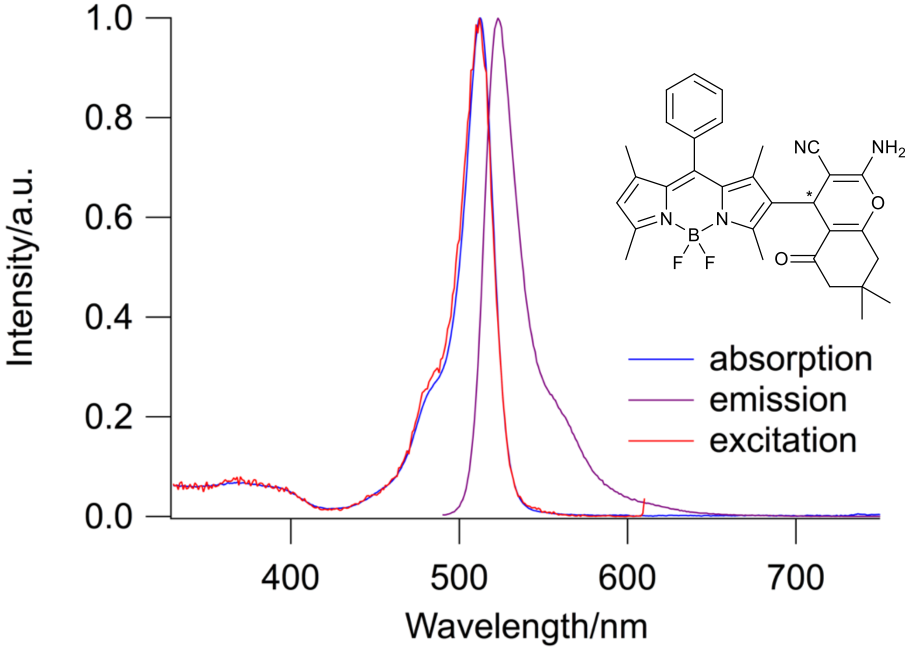

Figure S13. Absorption, fluorescence (λ_{ex} = 485 nm) and fluorescence excitation spectra (λ_{em} = 615 nm) of compound **4** in DCM.

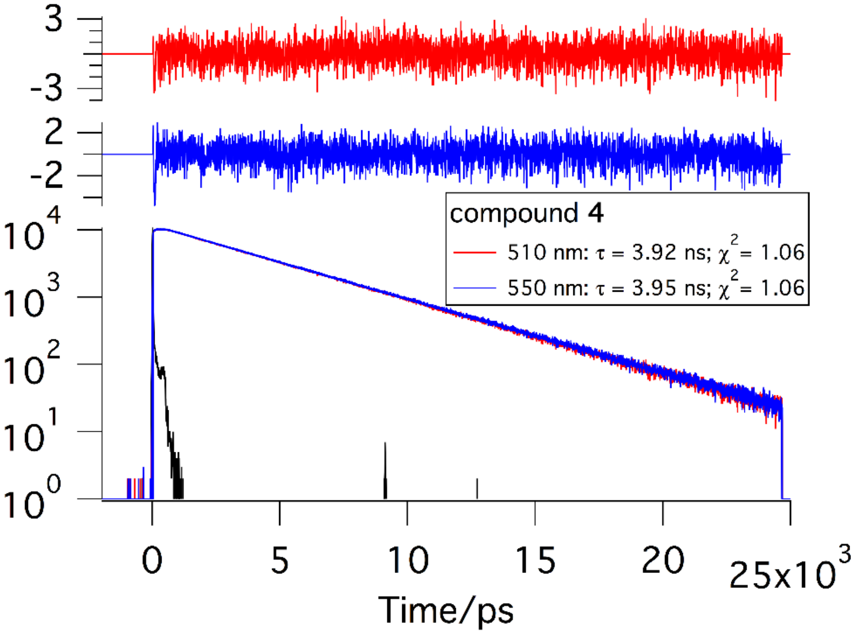

Figure S14. Time resolved fluorescence of compound **4** at two different emission wavelengths in DCM. IRF shown in black.
